# MRI Findings of Syndrome of Acute Bilateral Symmetrical Basal Ganglia Lesions in Diabetic Uremia: A Case Report and Literature Review

**DOI:** 10.1155/2016/2407219

**Published:** 2016-07-17

**Authors:** Xin Cao, Qiang Fang, Hao Shi

**Affiliations:** ^1^Medical College, Shandong University, Jinan, Shandong 250012, China; ^2^Medical College, Taishan Medical University, Tai'an, Shandong 271000, China; ^3^Department of Medical Imaging, Qianfoshan Hospital Affiliated to Shandong University, Jinan, Shandong 250014, China

## Abstract

The syndrome of acute bilateral basal ganglia lesions is an uncommon clinical occurrence exhibiting acute onset of movement abnormalities, which can be seen almost exclusively among patients with chronic renal failure, especially in the setting of concurrent diabetes mellitus. Symmetrical lesions located in basal ganglia demonstrated in MRI are typical manifestation of this syndrome. Our study includes routine MRI examination, MRS, 3D-ASL, and SWI findings, which have been rarely reported and will contribute to diagnosing more cases about this syndrome.

## 1. Introduction

The syndrome of acute bilateral basal ganglia lesions is an illness which has been rarely reported so far. It is Wang and his colleagues who first found this syndrome in three patients back in 1998 [1]. Patients with this syndrome often present with several typical clinical symptoms including gait disturbance, dysarthria, and Parkinson-like tremor. Bilateral basal ganglia lesions were found in an Asian patient presented in this study suffering from end-stage diabetic nephropathy on dialysis and showing clinical signs of gait disturbance, dysarthria, and involuntary movements.

## 2. Case Report

**Figure 1:**
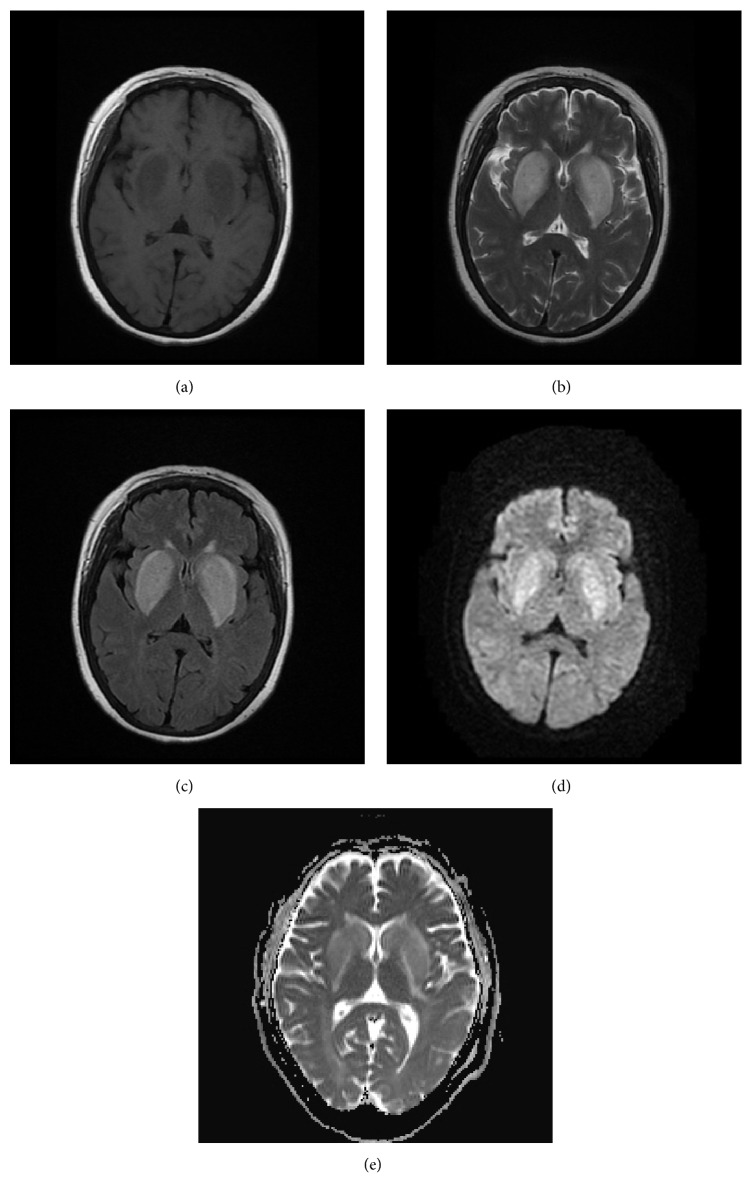
(a) Axial brain MRI showed symmetric hypointensity in the bilateral basal ganglia on T1-weighted images. (b, c) Axial brain MRI showed symmetric hyperintensity in the focal region on T2-weighted images and FLAIR images. (d) Diffusion-weighted imaging revealed slightly higher inhomogeneous signals in the involved regions. (e) ADC map revealed that the ADC value of the involved regions is not low.

**Figure 2:**
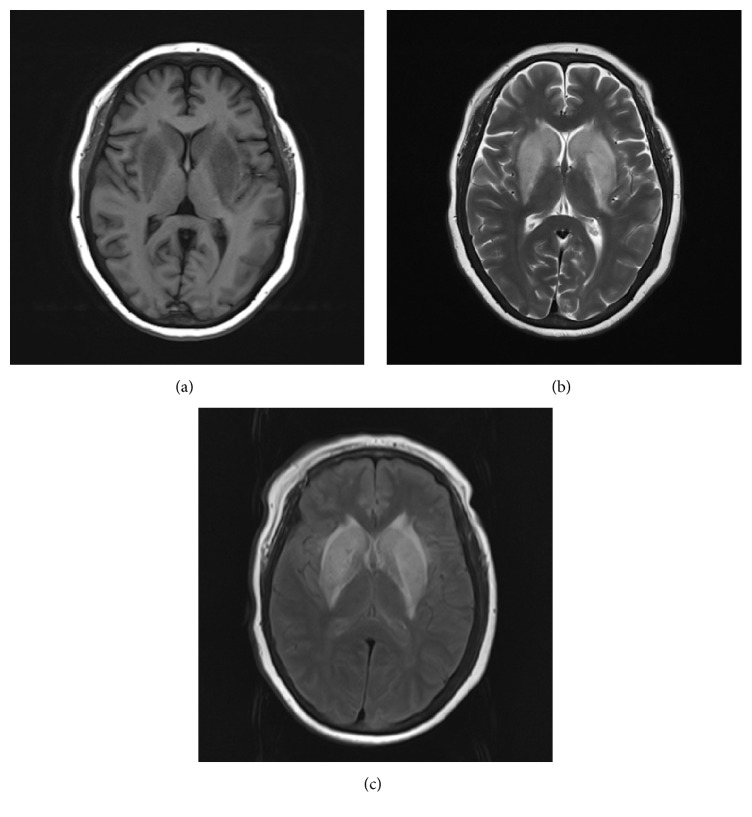
(a, b) The lesions extended in both T1WI and T2WI after 3 weeks. (c) FLAIR images showed demyelination around bilateral ventricles and lacunar infarctions.

**Figure 3:**
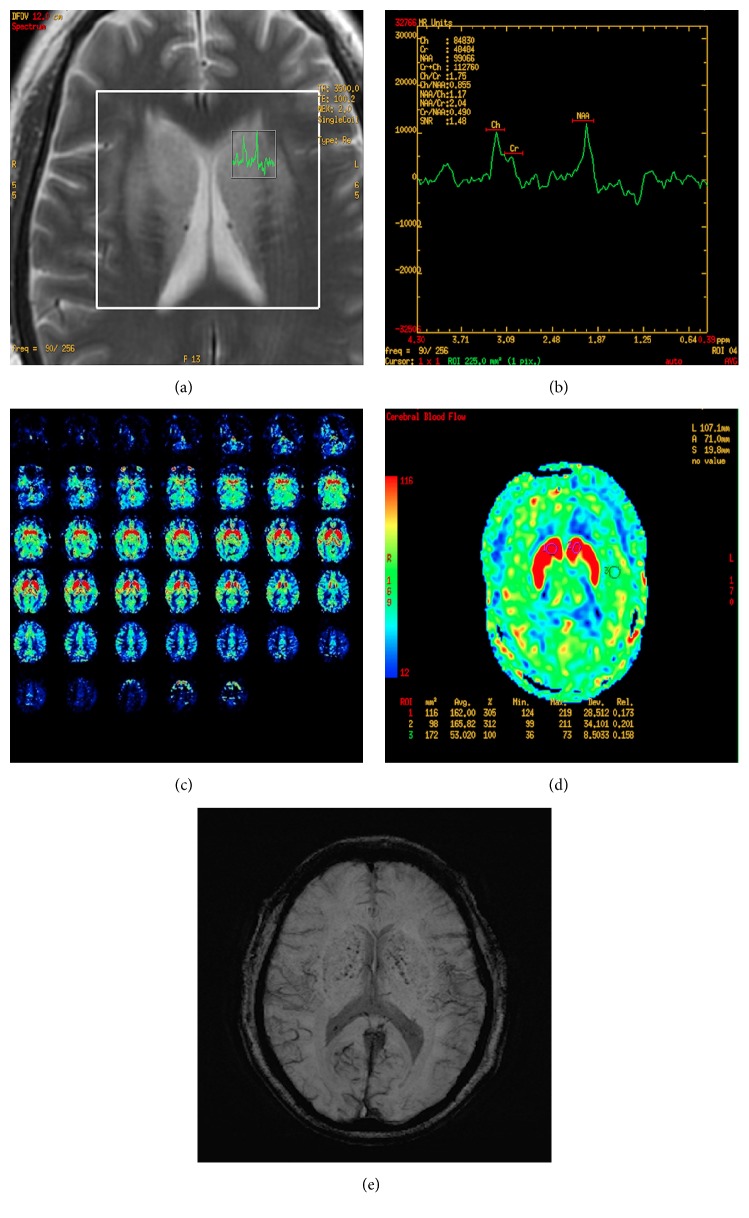
(a, b) MRS with region of interest pinpointed on the left basal ganglia lesion displayed a decrease of NAA/Cr value (2.04) and lactate doublet was detected. (c, d) 3D-ASL showed hyperperfusion in the lesion area and the rCBF was more than 160 mL/(100 g*∗*min). (e) SWI findings indicated old microbleeds and hemosiderin deposition.

A 49-year-old Chinese female was admitted to the hospital with gait disturbance, dysarthria, and involuntary movement of limbs for 10 days, with a history of edema in both lower extremities for 10 years, hypertension for almost 8 years, and diabetes mellitus for 2 years. Seven years ago when her serum creatinine level reached 2300 *μ*mol/L, she began taking regular hemodialysis treatments twice a week and then three years later this increased to three times a week due to her restless legs syndrome (RLS) secondary to chronic renal failure. Besides, she had polycystic kidney disease and hyperparathyroidism. There was no previous history of movement disorders and family history of this kind. Clinical examination revealed her highest blood pressure to be 200/100 mmHg. Generalized chorea was noted mainly on the extremities. Laboratory results showed elevated concentrations of blood urea nitrogen (BUN) of 33.6 mg/dL and creatinine (Cr) of 5.4 mg/dL, serum potassium of 6.1 mmol/L, and parathyroid hormone of 1457 pg/mL. Axial brain MRI revealed symmetric edema in the bilateral basal ganglia, which exhibited hypointensity on T1-weighted images (Figure 1(a)) and hyperintensity on both T2-weighted images (Figure 1(b)) and fluid-attenuated inversion recovery (FLAIR) images (Figure 1(c)). Diffusion-weighted imaging (DWI) demonstrated slightly higher inhomogeneous signals in the involved regions (Figure 1(d)). Concurrently, increased apparent diffusion coefficient (ADC) values were demonstrated in the periphery compared with normal brain tissue (Figure 1(e)), indicating vasogenic edema rather than cytotoxic edema. Follow-up MRI of brain was performed 3 weeks later, which showed lesions worsened and extended in both T1WI (Figure 2(a)) and T2WI (Figure 2(b)). Simultaneously, MR imaging revealed demyelination around bilateral ventricles and lacunar infarctions in FLAIR (Figure 2(c)). MR angiography showed atherosclerotic alterations of small brain vessels. Proton (1H) MR spectroscopy (MRS) (Figures 3(a) and 3(b)) with region of interest pinpointed on the left basal ganglia lesion displayed a decrease of N-acetylaspartate (NAA) peak and lactate (Lac) doublet. 3D-ASL results (Figure 3(c)) reflected the local perfusion condition, and the increased regional cerebral blood flow (rCBF) suggested hyperperfusion in the corresponding lesion area. Some dotted low signals were noted in the susceptibility weighted imaging (SWI) findings (Figure 3(e)). After two months, we were surprised to find that the abnormal signals of the lesions totally disappeared when we scanned her again, and this demonstrated that the acute bilateral basal ganglia lesions are reversible. The reports have been approved by the Institutional Review Board with patient informed consent.

## 3. Discussion

Acute movement disorders associated with isolated bilateral basal ganglia lesions have been recognized in patients with end-stage renal disease, particularly in the setting of diabetic nephropathy. Of the 24 cases reviewed by Li et al. [2], gait disturbance (76%), dysarthria (71%), and bradykinesia (47%) were the most common neurologic features. Tremor and rigidity, which are typical of parkinsonism, were noted in only 19% and 38% of all patients, respectively [2]. The neuroradiological abnormalities of patients with uremic encephalopathy are often seen in the cortical rather than bilateral basal ganglia regions [3]. Symmetric bilateral basal ganglia lesions on neuroimaging can be probably caused by carbon monoxide intoxication, hypoxia, toxins, metabolic disorders, small vessel vasculitis, or infection, but these lesions usually do not regress spontaneously [4]. Initial spontaneous improvement of the clinical symptoms and regression of the neuroimaging changes in this syndrome are common [5]. However, in this case, the condition of our patient worsened after three weeks owing to her poor long-term prognosis. Therefore, we believe that the chances of recovery are closely related to the treatment of diabetic uremia.

DWI can be used to identify whether the bilateral ganglia lesions are caused by vasogenic edema or cytotoxic edema. In this case, the ADC maps revealed increased water mobility in the involved regions, which indicates that the edema in this syndrome is not cytotoxic in nature, but increased interstitial fluid from breakdown of autoregulation, and we held similar views to Lee et al. [6]. Nevertheless, some small regions of cytotoxic edema can also be detected in the central parts by DWI. These features, observed also by Kim and his colleagues, indicate that some regions in the basal ganglia lesions are undergoing irreversible cytotoxic damage [7]. The basal ganglia especially globus pallidus have sufficient mitochondrion and blood supply and are susceptible to a wide range of toxins and metabolic changes. The vasogenic edema caused by worsening renal condition and long-term diabetes led to the opening of endothelial tight junctions, autoregulatory dysfunction in small vessels, and disturbance of tissue homeostasis. Thus, being exposed to uremic toxins for a long time will be detrimental to the basal ganglia.

Proton (1H) MR spectroscopy showed a declining NAA peak and a rising lactate peak inside the lesions, which suggests decreased neuron and glucose utilization failure. Our findings coincided with the research of Dicuonzo et al. [8]. In addition, reduced uptake of glucose in the bilateral basal ganglia was demonstrated in two cases by Wang et al., on 18F-fluorodeoxyglucose positron emission tomography (FDG-PET) examination, and the energy use failure is considered to be either dysregulation of the cerebral circulation or lower brain cell activity [9].

3D-ASL findings in the syndrome of acute bilateral basal ganglia lesions have never been reported to date. We found that the rCBF was more than 160 mL/(100 g*∗*min) in bilateral basal ganglia lesions regions, while the rCBF was less than 60 mL/(100 g*∗*min) in normal cerebral tissue (Figure 3(d)). Our results were inconsistent with Lin [3] who deemed that the pathophysiological mechanism was acute cerebral hypoperfusion. Whether the perfusion condition remains unchanged or fluctuated since the acute phase is unknown. Patients with this syndrome may have microangiopathic changes and endothelium-dependent dysfunction, which will further induce focal destructive endothelial lesions and the breakdown of blood brain barrier [6]. Under these circumstances, the blood perfusion will likely increase and the rCBF will even be overestimated. Moreover, when the basal ganglia were further exposed to highly elevated uremic or metabolic toxins, the regional cellular metabolism may have been disturbed, or functional disturbance in smooth muscle cells of vessels of the basal ganglia may have been induced, which will ultimately lead to vasodilatation and focal hyperemia [6].

Susceptibility weighted imaging (SWI) is based on the blood oxygen level dependent effect, and it is exquisitely sensitive to paramagnetic substances, such as deoxygenated blood, blood products, iron, and calcium. Some dotted low signals were noted in the bilateral basal ganglia lesions regions, which indicated old microbleeds and hemosiderin deposition instead of calcifications. It may be related to endothelial cell injury and cerebral tiny vessel hemorrhage associated with diabetes mellitus. Focal hyperemia and vascular atherosclerosis may also increase permeability of blood brain barrier.

There are some other theories proposed by other researchers lacking solid evidence. For example, Sheu et al. assumed that changes in hemodynamics caused by hemodialysis are the primary reason for this injury [10]. Park et al. thought that thiamine deficiency can lead to cellular hypoxia and extrapyramidal motor dysfunction due to the blocking of the citric acid cycle [11]. Anyhow, the etiology of such lesions must be multifactorial.

In conclusion, we report the first patient with syndrome of acute bilateral basal ganglia lesions of SWI and 3D-ASL. Although the definitive treatment is uncertain, it is imperative to correct uremic toxins and metabolic derangement in time. The enhanced awareness on this field and the development of neuroimaging will gradually form a full picture of this syndrome.
